# A Real-World Observation of Eltrombopag and Recombinant Human Thrombopoietin (rhTPO) in Lymphoma Patients With Chemotherapy Induced Thrombocytopenia

**DOI:** 10.3389/fonc.2021.701539

**Published:** 2021-08-18

**Authors:** Qiuhua Zhu, Shimei Yang, Wenbin Zeng, Mingjie Li, Zebing Guan, Lanlan Zhou, Hong Wang, Yanan Liu, Yanmin Gao, Shiqiu Qiu, Chaolun Chen, Huan Li, Shanshan Zheng, Yuemei Yuan, Hanling Zhang, Xueyi Pan

**Affiliations:** Department of Hematology, The First Affiliated Hospital of Guangdong Pharmaceutical University, Guangdong, China

**Keywords:** eltrombopag, recombinant human thrombopoietin (rhTPO), lymphoma, chemotherapy, thrombocytopaenia

## Abstract

This real-world, observational study aimed to assess and compare the clinical efficacy and safety of eltrombopag with recombinant human thrombopoietin (rhTPO) in the treatment of chemotherapy induced thrombocytopenia (CIT) in patients with lymphoma. One hundred and fifty-three patients who experienced grade 3 or 4 thrombocytopenia after chemotherapy for lymphoma were enrolled, 51 of which were treated with eltrombopag, 50 with rhTPO, and 52 patients with no drug treatment were served as the control group. The lowest platelet level and mean platelet counts at Day 5, Day 7, and Day 10 were significantly higher in both the eltrombopag group (P=.041,.003,.000,.000) and rhTPO group (P=.005,.005,.000,.000) than the control, but there was no difference between treatment with eltrombopag and rhTPO. Similarly, days required for the recovery of platelet counts to ≥50×10^9^/L and ≥75×10^9^/L were not different between the two treatment groups but significantly higher than the control group (P <.05). Rates of bleeding and platelet transfusion were all significantly reduced in patients treated with eltrombopag (P=.031,.032) or rhTPO (P=.017,.009) when compared to the control. Treatment-related adverse events (AEs) were reported in 7 (13.7%) and 6 (12.0%) patients in the eltrombopag and rhTPO groups, respectively, all being mild and transient in nature. In conclusion, both eltrombopag and rhTPO were effective and safe in the treatment of thrombocytopenia after chemotherapy for lymphoma.

## Introduction

Lymphoma is a malignant tumor originating from lymph nodes and (or) extranodal lymphoid tissues. The main symptoms include painless lymphadenopathy, hepatosplenomegaly, accompanying whole-body and multi-organ reactions such as fever, drenching night sweat, wasting, and itching (1). Myelosuppression, presented as different degrees of leukopenia, thrombocytopenia and anemia, is prevalent in patients with lymphoma who are undergoing chemotherapy (2). Chemotherapy-induced thrombocytopenia (CIT) not only increases the risk of hemorrhage, but also causes chemotherapy dose delays, dose reductions, or even treatment discontinuation that may result in prolonged hospitalization, increased medical costs, and reduced progression-free and overall survival (3–5).

At present, the treatment of CIT for lymphoma is very limited, which mainly include platelet transfusion and recombinant human interleukin-11 (rhIL-11). Platelet transfusion is the fastest and most effective treatment for severe CIT; however, as a rescue therapy, it is associated with temporary effectiveness, adverse reactions, and loss of response with repeated administrations (3, 6). rhIL-11 is the only platelet growth factor approved by the United States (US) Food and Drug Administration (FDA) and China National Medical Products Administration (NMPA) for CIT, but its use is severely restricted by the narrow therapeutic index and significant side effects including edema and arrhythmia (7). Therefore, novel treatment options are needed to improve the efficacy and safety outcomes of thrombocytopenia after chemotherapy for lymphoma.

Thrombopoietic agents were designed to stimulate the c-mpl receptor that leads to megakaryocyte maturation and platelet production (7). The rhTPO is a full-length glycosylated thrombopoietin prepared from Chinese hamster by recombinant DNA technology, which has a pharmacological effect similar to endogenous thrombopoietin (TPO) (8). It is the only thrombopoietin receptor agonist (TPO-RA) that receives market approval in China for the treatment of CIT (9, 10). Eltrombopag, as a chemically synthesized, orally available, small molecule, nonpeptide TPO-RA, has been approved for the treatment of chronic immune thrombocytopenia, hepatitis C virus­related thrombocytopenia, and (refractory) severe aplastic anemia. Recent studies showed that eltrombopag played an anti-proliferative effect in hematologic malignancies, raising the possibility of its use in CIT treatment (11).

It was reported in several randomized, placebo-controlled phase I and II clinical trials that eltrombopag has achieved objective curative effect on thrombocytopenia during chemotherapy cycles, especially in advanced solid tumor patients receiving gemcitabine−based chemotherapy and patients with acute leukemia (6, 12–14). Currently, there’s no study that assessed TPO-RAs for treatment of CIT in patients with lymphoma, and it is unknown whether the two TPO-RAs, rhTPO and eltrombopag, are comparable with regard to the clinical effectiveness and tolerability in CIT treatment. Using the data from a tertiary clinical practice in China, this large sample, observational study innovatively reviewed and compared the efficacy and safety profiles of eltrombopag to the market-approved rhTPO in the treatment of thrombocytopenia after chemotherapy for lymphoma.

## Materials and Methods

This retrospective study was approved by the Ethics Committee of the First Affiliated Hospital of Guangdong Pharmaceutical University. Between April 2017 and September 2020, patients with histopathologically confirmed lymphoma who also experienced grade 3 or 4 thrombocytopenia (platelet counts <30×10^9^/L) after chemotherapy (15) at the Hematology Department of our institution were enrolled. For the same patient who met the above criteria in more than one chemotherapy cycles, data of the earliest occurrence of PLT < 30 ×10^9^/L that were treated either with eltrombopag, rhTPO, or no drug treatment were included in this study. Exclusion criteria included tumors secondary to lymphoma and concomitant hemophagocytic syndrome. Patients with documented treatment history of eltrombopag or rhTPO after chemotherapy for lymphoma in other hospitals, concurrent treatment with both eltrombopag and rhTPO, eltrombopag and rhIL-11, or rhTPO and rhIL-11, were also excluded. The need for informed consent was waived by the committee.

One hundred and fifty-three lymphoma patients (90 males, 63 females) with CIT were consecutively enrolled in this study. Among them, 51 patients were treated with eltrombopag, 50 patients were treated with rhTPO, and 52 patients without drug treatment were selected as controls. Eltrombopag or rhTPO was initiated when platelet counts fell below 30×10^9^/L after chemotherapy. The starting dose of eltrombopag was 50 mg daily. The dose was adjusted to 75 mg/day when the platelet counts decreased to less than 10×10^9^/L. For patients who received rhTPO, daily injection of 15,000 U was given subcutaneously. In all patients, platelet transfusion was initiated when the platelet level was less than 20×10^9^/L. If platelets increased to more than 100×10^9^/L, or 50 ×10^9^/L more than the baseline, treatment of eltrombopag or rhTPO was stopped.

Demographic, socioeconomic, and clinical characteristics were obtained from patients. Efficacy variables, including platelet counts at baseline and on day 3, 5, 7, 10, and the lowest platelet counts after treatment, days required for the recovery of platelet counts to ≥50×10^9^/L and ≥75×10^9^/L, respectively, duration of platelet counts <50×10^9^/L, platelet transfusion frequency and volume, and the modified World Health Organization (WHO) bleeding grades (10), were assessed. Adverse events (AEs) were monitored and assessed according to the Common Terminology Criteria for Adverse Events (CTCAE) Version 5.0 (15). Increased transaminases were defined as alanine aminotransferase (ALT) or aspartate aminotransferase (AST) ≥3×the upper limit of normal (ULN), and hyperbilirubinemia as total bilirubin ≥1.5×ULN.

### Statistical Analysis

All statistical analyses were performed using SPSS 19.0 (IBM Corp., Armonk, NY). Continuous variables were summarized by mean, standard deviation (SD), median, and range, while categorical variables by number and frequencies (%). Comparisons of continuous variables among the three groups were carried out using one-way analysis of variance (ANOVA) test continued with *post hoc* analysis (LSD-t test) or non-parametric test when data did not follow normal distribution or homogeneity of variance. Categorical variables were compared using chi-square tests. P-values <.05 (two-sided) were considered statistically significant.

## Results

### Demographics, Socioeconomics, and Baseline Clinical Characteristics

Median time of treatment was 8 (range 5-14) days for eltrombopag and 7 (range 3-14) days for rhTPO. Patients in the control group were followed for a median of 12 (range 7-17) days.

**Table 1** shows the demographic, socioeconomic, and clinical features by treatment cohorts. The three groups did not differ with regard to gender, age, and ethnicity. No significant difference was observed for socioeconomic variables including type of medical insurance, monthly household income, education level, and occupation (P>.05). Clinical characteristics that include BMI, ECOG score, type of lymphoma, disease duration, bone marrow (BM) invasion, chemotherapy regimen, radiation therapy, pre-chemotherapy platelets, leukocyte counts, hemoglobin, and previous bleeding were also similar among the three groups.

**Table 1:** Demographic, socioeconomic, and baseline characteristics.

	Eltrombopag (N=51)	rhTPO (N=50)	Control (N=52)	P value^a^
Male, n (%)	29 (56.9)	30 (60.0)	31 (59.6)	.941
Age (years),				
Mean ± SD	49.1 ± 18.7	50.8 ± 15.3	48.3 ± 18.2	.865
Range	15-86	25-87	17-80	
Ethnic group, n (%)				1.000^b^
Han	51 (100.0)	50 (100.0)	51 (98.1)	
Others	0 (0.0)	0 (0.0)	1 (1.9)	
Medical insurance, n (%)				.863^b^
Urban employees’ basic medical insurance	10 (19.6)	13 (26.0)	15 (28.8)	
Urban residents’ basic medical insurance	17 (33.3)	19 (38.0)	12 (23.1)	
New rural cooperative medical system	7 (13.7)	5 (10.0)	9 (17.3)	
Mixed medical insurance^c^	4 (7.8)	2 (4.0)	2 (3.8)	
Full coverage^d^	2 (3.9)	4 (8.0)	3 (5.8)	
Others^e^	3 (5.9)	3 (6.0)	4 (7.7)	
Uninsured	8 (15.7)	4 (8.0)	7 (13.5)	
Household income (¥/month)^f^, n (%)				.949^b^
<5000	18 (35.3)	14 (28.0)	19 (36.5)	
5000-10000	10 (19.6)	15 (30.0)	15 (28.8)	
10001-15000	6 (11.8)	8 (16.0)	5 (9.6)	
15001-20000	9 (17.6)	7 (14.0)	8 (15.4)	
>20000	5 (9.8)	3 (6.0)	3 (5.8)	
Unknown^g^	3 (5.9)	3 (6.0)	2 (3.8)	
Education level, n (%)				.606
Primary school or lower	17 (33.3)	15 (30.0)	22 (42.3)	
Middle school	14 (27.5)	13 (26.0)	10 (19.2)	
High school	12 (23.5)	15 (30.0)	9 (17.3)	
College or above	8 (15.7)	7 (14.0)	11 (21.2)	
Occupation, n (%)				.718
Manual workers	6 (11.8)	10 (20.0)	8 (15.4)	
Agricultural workers	9 (17.6)	6 (12.0)	10 (19.2)	
Self-employed	14 (27.5)	8 (16.0)	9 (17.3)	
Managers and professionals	10 (19.6)	11 (22.0)	14 (26.9)	
Unemployed	12 (23.5)	15 (30.0)	11 (21.2)	
BMI (kg/m^2^), mean (range)	21.1 (17.5-26.6)	21.0 (17.1-26.1)	20.6 (15.4-27.9)	.521
ECOG score, mean (range)	2.9 (2-4)	2.7 (2-4)	2.9 (2-4)	.063
Time since diagnosis of lymphoma (months), mean (range)	16.0 (1-120)	18.0 (1-60)	14.0 (1-72)	.856
Type of lymphoma, n (%)				1.000^b^
DLBCL	28 (54.9)	26 (52.0)	28 (53.9)	
T or B lymphoblastic lymphoma	7 (13.7)	7 (14.0)	8 (15.4)	
NK/T-cell lymphoma	5 (9.8)	5 (10.0)	4 (7.7)	
PTCL	3 (5.9)	4 (8.0)	4 (7.7)	
Burkitt lymphoma	6 (11.8)	6 (12.0)	5 (9.6)	
Other types^h^	2 (3.9)	2 (4.0)	3 (5.8)	
Bone marrow invasion of lymphoma, n (%)	5 (9.8)	5 (10.0)	6 (11.5)	.952
Chemotherapy, n (%)				1.000^b^
CHOP or CDOP ± R	23 (45.1)	22 (44.0)	25 (48.1)	
R-EPOCH	3 (5.9)	3 (4.0)	3 (5.8)	
R-CODOX-M or R-IVAC	6 (11.8)	6 (14.0)	5 (7.7)	
CAM	3 (5.9)	4 (8.0)	4 (7.7)	
VDLP	4 (7.8)	3 (6.0)	4 (7.7)	
P-Gemox	5 (9.8)	5 (10.0)	4 (9.6)	
DICE	4 (7.8)	4 (8.0)	3 (5.8)	
Other chemotherapy^i^	3 (5.9)	3 (6.0)	4 (7.7)	
Prior chemotherapy regimens, mean (range)	6.1 (1-20)	6.6 (1-19)	6.9 (1-16)	.518
Radiation therapy, n (%)	3 (5.9)	3 (6.0)	2 (3.9)	.860
Platelet count before chemotherapy (×10^9^/L)				
Mean ± SD	135.7 ± 129.1	130.3 ± 78.7	134.7 ± 87.1	.512
Range	5-625	7-309	8-370	
Leukocyte count (×10^9^/L)				
Mean ± SD	2.5 ± 2.7	2.5 ± 2.5	2.6 ± 3.5	.469
Range	0.14-11.19	0.05-12.11	0.08-16.54	
Hemoglobin (g/L)				
Mean ± SD	75.0 ± 18.0	78.7 ± 17.7	78.7 ± 22.7	.457
Range	44-138	45-115	40-149	
Previous bleeding^j^, n (%)	2 (3.9)	3 (6.0)	2 (3.8)	.798

BMI, body mass index; CAM, cyclophosphamide, cytarabine, mercaptopurine; CDOP ± R, (cyclophosphamide, vincristin, prednisone, adriamycin/liposomal adriamycin) ± rituximab; DICE, dexamethasone, ifosfamide cisplatin etoposide; DLBCL, diffuse large B-cell lymphoma; ECOG, Eastern Cooperative Oncology Group; NK/T-cell lymphoma, Natural killer/T-cell lymphoma; P-Gemox, peaspartase, gemcitabine, oxaliplatin; PTCL, peripheral T-cell lymphoma; R-CODOX-M, tuximab, cyclophosphamide, vincristine, adriamycin, methotrexate; R-IVAC, tuximab, ifosfamide, etoposide, arabinoside glycoside; VDLP, prednisone, daunorubicin or liposomal adriamycin, vincristine, pegaspase.

a P value compares baseline characteristics among three groups.

b Using Fisher’s exact test in R*C table Chi-Square Test.

c Two or more insurances, normally indicating the basic insurance plus other supplementary insurances in China.

d Medical costs can be almost fully covered.

e Other single insurance.

f ¥5000 equals to US$775, ¥5000-10000 equals to US$775-1550, ¥10001-15000 equals to US$1550-2325, ¥15001-20000 equals to US$2325-3100, ¥20000 equals to US$3100.

g Patients either refused to answer the question or did not know the answer.

h Other types of lymphoma include marginal area lymphoma, follicular lymphoma and mantle cell lymphoma.

i Other chemotherapy include GDP (gemcitabine, cisplatin, dexamethasone), BR (bendamustine, rituximab), and R+MTX (rituximab, methotrexate).

j WHO bleeding grades 1 or 2.

### Treatment Efficacy

#### Platelet Response

**Figure 1:**
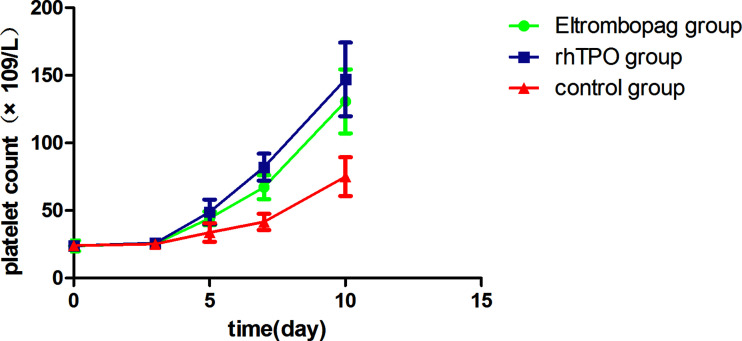
Mean platelet counts during treatment period among the three groups. Error bars indicate 95% CI of the mean. CI, confidence interval.

Mean platelet counts at baseline (Day 0) were not different among the three groups [eltrombopag: (23.96 ± 14.15) ×10^9^/L, rhTPO: (23.92 ± 12.45) ×10^9^/L, control: (24.15 ± 7.47) ×10^9^/L; P=.711]. At Day 5, Day 7, and Day 10, significantly higher platelet counts were observed in both the eltrombopag group [(44.24 ± 17.51) ×10^9^/L, (67.30 ± 29.90) ×10^9^/L, (130.73 ± 70.57) ×10^9^/L; P=.003,.000,.000] and rhTPO group [(48.92 ± 32.46) ×10^9^/L, (82.11 ± 33.37) ×10^9^/L, (147.02 ± 68.47) ×10^9^/L; P=.005,.000,.000] than the control group [(33.73 ± 24.62) ×10^9^/L, (41.58 ± 21.27) ×10^9^/L, (75.67 ± 40.40) ×10^9^/L], but there was no difference between treatment with eltrombopag and rhTPO (P=1.000,.187,.598; **Table 2** and **Figure 1**). Similar trend was reflected in the lowest platelet count, which was significantly lower in the control group than the eltrombopag group and the rhTPO group [(11.37 ± 7.66) ×10^9^/L *vs* (15.94 ± 9.09) ×10^9^/L, (18.28 ± 15.59) ×10^9^/L; P=.041,.005], but no difference was seen between the two treatment groups (P=1.000).

**Table 2:** Platelet counts among the three groups.

	Eltrombopag (A)	rhTPO (B)	Control (C)	P value			
				Overall^a^	A *vs* C	B *vs* C	A *vs* B
Day 0, N	51	50	52				
Platelet count (×10^9^/L)	23.96 ± 14.15	23.92 ± 12.45	24.15 ± 7.47	.711	/	/	/
Day 3, N	51	50	52				
Platelet count (×10^9^/L)	25.24 ± 11.12	25.94 ± 10.08	25.21 ± 5.70	.613	/	/	/
Day 5, N	51	50	52				
Platelet count (×10^9^/L)	44.24 ± 17.51	48.92 ± 32.46	33.73 ± 24.62	.001	.003	.005	1.000
Day 7, N	46	45	50				
Platelet count (×10^9^/L)	67.30 ± 29.90	82.11 ± 33.37	41.58 ± 21.27	.000	.000	.000	.187
Day 10, N	44	44	45				
Platelet count (×10^9^/L)^b^	130.73 ± 70.57	147.02 ± 68.47	75.67 ± 40.40	.000	.000	.000	.598
The lowest platelet count (×10^9^/L)^b^	15.94 ± 9.09	18.28 ± 15.59	11.37 ± 7.66	.004	.041	.005	1.000
P1^c^	0.614	0.375	0.419				
P2^d^	0.000	0.000	0.113	/	/		/
P3^e^	0.000	0.000	0.000	/	/		/
P4^f^	0.000	0.000	0.000	/	/		/

Data are mean ± SD.

a P value compares platelet counts among the three groups.

b Defined as the lowest platelet count observed after treatment initiation (or after enrolment for patients of the control group).

c P value compares platelet counts between Day 0 and Day 3.

d P value compares platelet counts between Day 0 and Day 5.

e P value compares platelet counts between Day 0 and Day 7.

f P value compares platelet counts between Day 0 and Day 10.

Platelet counts <50×10^9^/L lasted for 6.25 ± 2.61 days in the eltrombopag group and 5.48 ± 2.62 days in the rhTPO group (P=.599), both of which were significantly shorter than the control group [(8.33 ± 3.98) days; P=.036,.000]. Days required for the recovery of platelet counts to 50×10^9^/L or higher was comparable between patients treated with eltrombopag and those with rhTPO (P=.508) but significantly shorter than the control group [(6.33 ± 2.31) days, (5.44 ± 2.57) days *vs* (8.32 ± 2.53) days; P=.001,.000]. The findings were similar with regard to the days required for the recovery of platelet counts to ≥75×10^9^/L (**Table 3**).

**Table 3:** Platelet response among the three groups.

	Eltrombopag (A)	rhTPO (B)	Control (C)	P value			
				Overall^a^	A *vs* C	B *vs* C	A *vs* B
Platelet count <50×10^9^/L, N	51	50	52				
Lasting days	6.25 ± 2.61	5.48 ± 2.62	8.33 ± 3.98	.001	.036	.000	.599
Platelet count ≥50×10^9^/L, N	48	48	50				
Required days	6.33 ± 2.31	5.44 ± 2.57	8.32 ± 2.53	.000	.001	.000	.508
Platelet count ≥75×10^9^/L, N	46	46	47				
Required days	7.43 ± 2.54	6.56 ± 3.78	9.61 ± 2.55	.000	.004	.000	.451

Data are mean ± SD.

a P value compares data among the three groups.

#### Bleeding Outcomes

**Figure 2:**
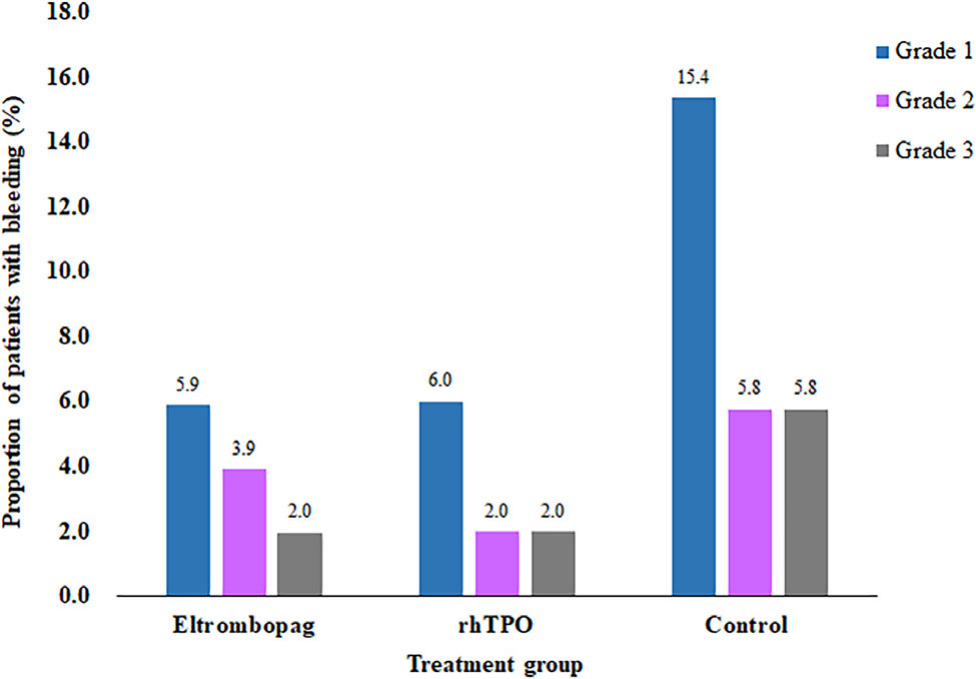
Proportion of bleeding by WHO grades among the three groups.

WHO grade 1 bleeding occurred in 3 patients (5.9%) treated with eltrombopag, 3 patients (6.0%) with rhTPO, and 8 (15.4%) patients in the control group, whereas grade 2 or 3 bleeding occurred in 3 (5.9%) patients treated with eltrombopag, 2 (4.0%) patients with rhTPO, and 6 (11.5%) patients in the control group (**Figure 2**). No cases of grade 4 bleeding occurred during study. Rates of overall bleeding (any grades 1-4) were significantly higher in the control group than eltrombopag and rhTPO groups (26.9% *vs* 11.8%, 10.0%; P=.031,.017), yet there was no statistical difference between treatment with eltrombopag and rhTPO (P=.776; **Table 4**).

**Table 4:** Bleeding by WHO grades and platelet transfusion after treatment.

	Eltrombopag (A)	rhTPO (B)	Control (C)	P value			
	**(N=51)**	**(N=50)**	**(N=52)**	Overall^a^	**A *vs* C**	**B *vs* C**	**A *vs* B**
Any bleeding (Grades 1-4), n (%)	6 (11.8)	5 (10.0)	14 (26.9)	.019	.031	.017	.776
Platelet transfusion, n (%)	28 (54.9)	25 (50.0)	39 (75.0)	.023	.032	.009	.622

a P value compares data among the three groups.

#### Platelet Transfusion

A total of 28 (54.9%), 25 (50.0%), and 39 (75.0%) patients received one unit of platelet transfusion in the eltrombopag group, the rhTPO group, and the control group, respectively. Compared to the two treatment groups, a significantly higher proportion of cases required platelet transfusion in the control group (P<.05; **Table 4**). Platelet transfusion rates did not differ between eltrombopag and rhTPO treatment (P=.622).

### Safety and Tolerability

Seven patients (13.7%) experienced AEs that may be related to eltrombopag treatment, including 3 cases of elevated transaminase (5.9%) and 1 case each (2.0%) of hyperbilirubinemia, fever, fatigue, and dizziness. Six patients (12.0%) experienced AEs that may be related to rhTPO treatment, including 2 cases of fever (4.0%) and 1 case each (2.0%) of fatigue, dizziness, diarrhea, and muscle aches. All these AEs were mild and transient in nature. The elevation of transaminase and blood bilirubin in the eltrombopag group were resolved after a short-term liver protection treatment, while the other AEs were relieved spontaneously without special treatment. No serious AEs were reported. Overall, eltrombopag and rhTPO were well tolerated in our study (**Table 5**).

**Table 5:** Treatment-related adverse events.

	Eltrombopag (N=51)	rhTPO (N=50)
Any AE, n (%)	7 (13.7)	6 (12.0)
Elevated transaminase	3 (5.9)	0 (0.0)
Hyperbilirubinemia	1 (2.0)	0 (0.0)
Fever	1 (2.0)	2 (4.0)
Fatigue	1 (2.0)	1 (2.0)
Dizziness	1 (2.0)	1 (2.0)
Diarrhea	0 (0.0)	1 (2.0)
Muscle aches	0 (0.0)	1 (2.0)

## Discussion

In China, rhTPO was recommended for the treatment of severe CIT and as a prophylactic option in cancer patients with high risk of bleeding after chemotherapy (9, 10). The efficacy and safety of rhTPO to treat CIT in patients with solid tumor have been well demonstrated in previous studies (16, 17). Eltrombopag was reported as an effective agent to maintain platelet level, reduce bleeding episodes and transfusion requirements, and alleviate chemotherapy dose reductions and delays without compromising patient safety in a number of small-sample, early phase trials (6, 12–14, 18–21). Due to limited data available, eltrombopag has not been approved for the treatment of CIT; however, in view of the difficulties of CIT treatment and seriousness of bleeding consequences, it is considered as an alternative therapeutic option for use in patients with poor response to rhTPO (10). This is the first study to systematically evaluate and compare the effectiveness and safety of eltrombopag *versus* rhTPO for treatment of CIT in patients with lymphoma. Based on a large cohort of patients, our findings provide summarized experience in a real-world clinical practice.

Cytotoxic drugs used in chemotherapy can lead to increased platelet destruction, reduced platelet production, and abnormal platelet distribution that result in thrombocytopenia (22). TPO-RAs were designed as mimics to endogenous TPO that regulates the whole process of megakaryopoiesis and promotes platelet production (3, 23, 24). rhTPO, as the first generation TPO-RA, was shown to be effective in alleviating and shortening the duration of CIT at a daily dose of 15,000 U in our patients with lymphoma. Eltrombopag is the second generation, non-peptide TPO-RA that binds to the transmembrane domain of the thrombopoietin receptor and increases platelet counts by stimulating megakaryocytes proliferation from BM progenitor cells (25). Unlike rhTPO, eltrombopag does not compete with or elicit an antibody response to endogenous TPO (26). At a starting dose of 50 mg and adjustable daily dose during treatment, our results indicated that eltrombopag was comparable to rhTPO in terms of the efficacy to increase platelet counts and nadir platelet counts, and reduce the time required for platelet recovery.

In a study of rhTPO for the treatment of CIT in patients with solid tumor, time required for platelet levels recovered to ≥75×10^9^/L and ≥100×10^9^/L were 4.79 ± 3.67 and 6.93 ± 3.61 days, respectively, among 72 patients who had a post-chemotherapy platelet count of 55.9 ± 16.0 (×10^9^/L) (27). Another randomized, cross-over, self-controlled trial of rhTPO reported a mean of 2.5 ± 3.9, 10.3 ± 8.7, and 15.9 ± 10.5 days with respect to the duration of thrombocytopenia (platelet counts <50×10^9^/L), days required for the recovery of platelet counts to ≥75×10^9^/L and ≥100×10^9^/L, respectively (17). Our findings on the days required for the recovery of platelet counts to ≥75×10^9^/L [eltrombopag (7.43 ± 2.54) days, rhTPO (6.56 ± 3.78) days; **Table 3**] were comparable to the aforementioned studies, yet the platelet counts <50×10^9^/L lasting days were observed to be longer in either of our treatment groups [eltrombopag (6.25 ± 2.61) days, rhTPO (5.48 ± 2.62) days; **Table 3**]. This might be explained by the presence of more severe thrombocytopenia at baseline in our patient population, as subgroup analysis of severe CIT in the study of Bai et al. (16) reported longer duration of platelet counts <50×10^9^/L (median 11 days) and that median time required for the recovery of platelet counts to ≥75×10^9^/L and ≥100×10^9^/L were 21 and 24 days, respectively. Due to the real-world nature of this study, some patients gave up the treatment due to financial burden such that we did not have adequate data to calculate days required for the recovery of platelet counts to ≥100×10^9^/L. Well controlled, prospective studies will be conducted in the future to observe platelet response in a longer period with more comprehensive follow-up schedules.

CIT increases the risk of bleeding that may lead to dose adjustment and delayed treatment schedules with chemotherapy (22, 28). It was studied in cancer patients after chemotherapy that when platelet counts fell below 50×10^9^/L, probability of any bleeding ranges between 0-9.6%; however, the risk doubles when platelet counts <20×10^9^/L (10.1-17.7%) and continues to double when <10×10^9^/L (18.4-40.1%) (7). Our study revealed that eltrombopag or rhTPO treatment was associated with significantly reduced rates of any bleeding and clinically significant bleeding (grade 2-4) when compared to the control group. In a study of patients with immune thrombocytopenia (ITP), regardless of platelet response, a reduced proportion of bleeding episodes were observed during eltrombopag treatment, which was considered to be correlated with the platelet adhesion effect enhanced by eltrombopag (29). Apart from a low baseline platelet count, a previous bleeding episode, BM metastasis, poor ECOG score (≥2), previous radiotherapy, and special chemotherapy regimen (cisplatin, carboplatin, gemcitabine, carmustine, or lomustine) were all related to elevated risk of bleeding in patients with CIT (30). Our findings showed no difference of bleeding rates between eltrombopag and rhTPO treatment groups; meanwhile, baseline predictors of bleeding were similar between the two groups, indicating that eltrombopag can be as effective as rhTPO with respect to reducing the risk and severity of bleeding events associated with CIT in patients with lymphoma.

Platelet transfusion is the elective procedure for prevention and treatment of bleeding in patients with hematological disorders, chemotherapy or hematopoietic stem cell transplantation. In case of an active bleeding, it is the first line of therapy if bleeding is considered associated with CIT (31). Prophylactic platelet transfusions are indicated when bleeding occurs or when platelet counts are <10×10^9^/L (or <20×10^9^/L if the patient is febrile) (3). However, repeated transfusions may lead to problems such as refractoriness, alloimmunization, febrile reactions, and transmission of infectious agents (7, 32, 33). Our study revealed significantly lower frequencies and volume of platelet transfusion in patients treated with eltrombopag or rhTPO when compared to the controls, but they were not different between eltrombopag and rhTPO treatment. When rhTPO was administered to patients with gynecologic cancer who developed severe thrombocytopenia after carboplatin chemotherapy, the need for platelet transfusion was reduced from the pre-treatment rate of 75% to 25% (P=.013) (34). Since it requires for about 5 days for platelet counts to rise after administering TPO-RAs, platelet transfusion, if indicated, should be used together with TPO-RAs (3). By stimulating platelet production that eventually leads to elevated platelet counts (24, 25), TPO-RAs such as eltrombopag and rhTPO can reduce the need for platelet transfusion due to bleeding or low platelet counts, thus lowering the overall risk of transfusion reactions and non-response.

In general, eltrombopag and rhTPO were well tolerated as all of the treatment-related AEs observed in our study were mild and in accordance with the safety profiles of previous reports (16, 19, 33, 35). Hepatobiliary toxicity is a major concern associated with eltrombopag use in patients of east Asian descent (20, 36). Elevated transaminase and hyperbilirubinemia occurred in 5.9% and 2.0% of our patients treated with eltrombopag, respectively. All these hepatobiliary abnormalities were mild and resolved after a short-term liver protection treatment. In a phase II study assessing the efficacy and safety of eltrombopag in patients receiving carboplatin/paclitaxel for treatment of advanced solid tumors, elevated aminotransferase ≥3 times the ULN and total bilirubin ≥1.5 times the ULN were 11% and 18% in the 50 mg group, 17% and 14% in the 75 mg group, and 13% and 23% in the 100 mg group (12). Hepatobiliary AEs were reported in 19% patients in a phase 1/2 trial assessing the safety and tolerability of eltrombopag for treatment of thrombocytopenia in patients with advanced myelodysplastic syndromes or acute myeloid leukemia, including 8% with grade 3 or higher events (14). Our rates and severity of elevated hepatobiliary values were lower than these findings, suggesting that eltrombopag can be safely prescribed to treat CIT in patients with lymphoma.

Due to the good tolerability and efficacy to reduce the incidence and duration of thrombocytopenia, rhTPO was proposed as a second-line treatment option for CIT by the Chinese Society of Clinical Oncology (9, 10). Our preliminary findings on a relatively large sample of patients with lymphoma suggested that eltrombopag had comparable efficacy and safety with rhTPO for treatment of CIT. Injection site reactions such as pain and ecchymosis are commonly expected in patients treated with rhTPO as the drug was approved for subcutaneous use. Eltrombopag, developed as an oral preparation, allows to avoid injection-related reactions and is considered more convenient for patients to administer. Well-designed medical cost research studies conducted in a wider geographic area of China are needed to further support if eltrombopag, when compared to rhTPO, is also a cost-effective treatment option to lymphoma patients with CIT.

There are several limitations in our study: first, because of retrospective and observational design, we are unable to evaluate how eltrombopag and rhTPO administration help to maintain scheduled dosing and treatment cycles of chemotherapy. Secondly, as this is a single-center study, generalizability of study results to other population should be made with caution. Thirdly, due to the inclusion of multiple chemotherapy regimens, it may not be possible to establish the impact of eltrombopag and rhTPO treatment on CIT after a specific regimen. Also, prognostic outcomes, such as progression-free and overall survival, cannot be explored owning to the relatively short duration of follow-up in the current study. Further prospective, interventional studies are needed to investigate the long-term effectiveness and safety of eltrombopag and rhTPO for treatment of CIT in patients with lymphoma that receive myelosuppressive chemotherapy, and to identify the optimal dose and dosing frequencies.

In conclusion, both eltrombopag and rhTPO were effective in the treatment of thrombocytopenia after chemotherapy for lymphoma with respect to the elevated platelet counts, prolonged periods of platelet response, reduced bleeding episodes and platelet transfusion requirements. Eltrombopag was well tolerated in real-world setting without raising additional concerns for hepatobiliary toxicity. Evidence from this large cohort study supports the use of eltrombopag as an alternative treatment option for CIT in lymphoma patients.

## Data Availability Statement

The original contributions presented in the study are included in the article/supplementary material. Further inquiries can be directed to the corresponding author.

## Ethics Statement

The studies involving human participants were reviewed and approved by Ethics Committee of the First Affiliated Hospital of Guangdong Pharmaceutical University. Written informed consent for participation was not required for this study in accordance with the national legislation and the institutional requirements.

## Author Contributions

QZ, SY, and XP conceptualized and designed the study. QZ and SY collected and analyzed data, and drafted the paper. LZ, WZ, and XP carried out the data analysis, and revised the paper. All authors contributed to the article and approved the submitted version.

## Funding

This study was supported by the Science and Technology Planning Project of Guangzhou (202002030253), the Natural Science Foundation of Guangdong Province (2017A030313664), the Science and Technology Project of Yuexiu District of Guangzhou (2017-W S-008).

## Conflict of Interest

The authors declare that the research was conducted in the absence of any commercial or financial relationships that could be construed as a potential conflict of interest.

## Publisher’s Note

All claims expressed in this article are solely those of the authors and do not necessarily represent those of their affiliated organizations, or those of the publisher, the editors and the reviewers. Any product that may be evaluated in this article, or claim that may be made by its manufacturer, is not guaranteed or endorsed by the publisher.
